# Investigation on the wind erosion resistance of aeolian sand solidified by enzyme mineralization combined with fiber reinforcement

**DOI:** 10.1038/s41598-025-18842-w

**Published:** 2025-09-29

**Authors:** Jia Liu, Lujing Yuan, Gang Li, Jing Qu, Jinli Zhang

**Affiliations:** 1 Shaanxi Key Laboratory of Safety and Durability of Concrete Structures, Xijing University, Xi’an, 710123 Shaanxi China; 2 State Key Laboratory of Coastal and Offshore Engineering, Dalian University of Technology, Dalian, 116024 China

**Keywords:** Aeolian sand, EICP, Fiber reinforcement, Wind erosion resistance, Model test, Biomaterials, Civil engineering

## Abstract

Sandstorms can lead to atmospheric pollution, soil degradation and health damage, which the origin of frequent outbreaks is that traditional methods cannot effectively solve the solidified of aeolian sand. Enzyme induced calcium carbonate precipitation (EICP) combined with basalt fiber reinforcement (BFR) or wool fiber reinforcement (WFR) method can significantly improved the strength and reduced the brittle fracture of sand. Based on the wind tunnel model test, this paper analyzed the effect of wind velocity (*v*), erosion angle (*α*), and erosion cycles (*N*) on the erosion resistance of aeolian sand solidified by EICP with BFR or WFR. According to analyzed the anti-erosion mechanism of aeolian sand, the erosion modulus model was established considered the effects of wind velocity and erosion angle. The results showed that compared with loose aeolian sand, EICP-solidified sand formed a hard layer on the surface, and the mass loss rate (*η*) increased with increasing of wind velocity, erosion angle and erosion cycles. Under the strongest erosion condition, the *η* of loose sand, EICP, EICP-BFR and EICP-WFR solidified aeolian sand reached 88.79%, 63.55%, 55.57% and 52.40%, respectively. As the number of erosion cycles increases, the *η* of EICP-solidified aeolian sand rises from 1.46 to 7.59%, that of EICP-BFR-solidified sand from 0.82 to 6.41%, and that of EICP-WFR-solidified sand from 0.71 to 6.26%. The addition of fiber can effectively promoted the cementation of CaCO_3_ crystal, improved the surface strength and wind erosion resistance, and reduced the quality loss of aeolian sand. The experimental results agreed well with the model prediction results, which validated the reliability of erosion modulus model. The research results can provide a guideline for aeolian sand solidified in desert area.

## Introduction

Sandstorms have the characteristics of strong suddenness, short duration, and great harm, which can lead to atmospheric pollution, soil erosion and degradation, and human health damage. Aeolian sand flow is the main cause of land desertification, and with the assistance of strong winds and local thermal instability, sandstorms are easily formed. Aeolian sand is the most abundant and inexpensive road building material in desert areas ^1^, with small and light particles, poor grading, low natural moisture con-tent, and difficult compaction. It is susceptible to serious environmental problems caused by wind erosion^2,3^. The formation of sandstorms requires three conditions: sustained and strong winds, unstable atmospheric stratification, and dry and loose sand material. The first two cannot be controlled or avoided, so solving the hazards of sand-storms requires a fundamental approach solidifying aeolian sand. However, traditional sand fixation methods are gradually unable to meet the requirements of low carbon, economy, efficiency, and environmental protection. Therefore, there is an urgent need for an effective sand fixation method to alleviate problems such as sandstorms and aeolian sand flow. Research^4^ has demonstrated that microbially induced calcium carbonate precipitation (MICP) technology effectively enhances the engineering performance of soft clays, with experimental results indicating that both vegetative bacterial cells and spores can promote strength and modulus improvements through the induction of calcite crystal formation. In contrast to MICP, which depends on viable bacterial activity and thus faces limitations in harsh desert environments, enzyme-induced carbonate precipitation (EICP) utilizes plant-derived urease, thereby ensuring superior stability under extreme temperature fluctuations and arid conditions.

It is evident that enzyme induced calcium carbonate precipitation (EICP) technology boasts a number of key advantages, including high efficiency, environmental friendliness, sustainability, and durability. This method has been applied in repairing heavy metal pollution^5–8^, restoring and protecting ancient buildings^9–11^, replacing certain civil engineering materials^12,13^, improving the thermal conductivity of backfill materials and soil^14^, suppressing dust^15^, and repairing high-temperature damaged concrete^16^. Alwalan et al.^17^ conducted direct shear tests on sand consolidated by EICP technology using four different methods of spraying, mixed compaction, infiltration, and injection for bonding samples. The present study found that the primary factors influencing the sand’s shear strength were related to the mechanism of action of EICP and the distribution position of CaCO_3_ precipitation in the sand. Almajed et al.^18^ used sodium alginate (SA) biopolymer assisted EICP to stabilize desert sand. As demonstrated by the findings of wind tunnel experiments, there is a direct correlation between the rate of erosion and the wind velocity. The erosion rate increases gradually as the wind velocity rises. When the concentration of biopolymer increased from 0.5 to 2.0%, the magnitude of the increase in strength exhibited by the sample that had undergone treatment with EICP-SA was found to be 36%. Martin et al.^19^ verified through unconfined compressive strength tests that the strength of EICP treated sand varies with the type of sand. The addition of skim milk powder to modify the EICP solution can sustainably increase the strength and bonding ability of EICP solutions for different types of sand. Recent studies on plant-derived enzyme-induced carbonate precipitation techniques—including soybean protein isolate-induced (SICP)^20^ and soybean urease-induced (SUICP)^21^ methods—have validated their eco-friendly efficacy in soil improvement. SICP enhances expansive soil strength and reduces compressibility with optimal performance at 1 mol/L cementation solution (64% unconfined compressive strength gain), while SUICP improves sand foundation bearing capacity by 45.09% via particle bonding and densification (though uniformity requires optimization). These align with broader EICP research^22^ showing significant soil property enhancements (e.g., 3 MPa strength, 98.2% permeability reduction) and highlight the need for standardized plant urease applications and long-term performance studies in special environments. Recent research^23^ on expansive clay stabilization showed that chemical additives like cement and lime effectively reduce swelling potential and enhance strength through hydration reactions, with microstructure analysis revealing particle cluster formation and pozzolanic products that correlate with improved mechanical properties. Ghasemi et al.^24^ studied the effects of different fiber types, such as polyester synthetic fibers (PES), basalt fibers (BS), and sugarcane bagasse natural fibers (BG), on the strength and mechanical behavior of sandy soil. Tensile strength tests were con-ducted with different fiber contents of 0.5%, 1%, 2%, and fiber lengths of 2.5 mm, 5 mm, and 7.5 mm as variables. The experimental results showed that the mechanical effect of the specimens reinforced with PES and BS fibers with a length of 7.5 mm and a content of 2% improved the best, with a 2.98-fold increase in tensile strength. Tang et al.^25^ conducted a consolidation drainage triaxial test on palm fiber-reinforced sandy soil using pure sand as the control group. The fiber length (8–20 mm) and fiber mass fraction (0.3% -0.9%) were used as variables to study the deviatoric stress, stress path, shear strength, volume change, porosity, and reinforcement coefficient of fiber-reinforced sandy soil. The test results showed that palm fibers increased the peak shear strength of sandy soil by about 10% -20%, and the increase in critical shear strength was greater than 100%. The fibers have a beneficial effect on the improvement of the shear strength of sand. The addition of fibers prolongs the axial strain required for sand to reach the critical porosity ratio, enhances the ability of sand to resist large deformations, and strengthens the toughness of sand. Research^26^ shows that studies on unidirectional jute fiber-reinforced polymer (UJFRP) for concrete confinement have demonstrated its superior tensile performance over conventional bidirectional jute FRP. UJFRP confinement significantly enhanced concrete compressive stress (up to 5.0x for low-strength, 1.7x for normal-strength concrete), with a simplified model accurately predicting compressive behavior, supporting its use as an eco-friendly retrofitting material. Beyond aeolian sand, similar stabilization principles have been validated in other problematic soils. Chen et al.^27^ conducted a triaxial consolidation undrained test to study the shear strength of basalt fiber-reinforced (BFR) loess. The test results showed that the peak strength of BFR loess increased with the increasing of fiber length, fiber content, and confining pressure, and decreased with the increasing of moisture content. Despite the fact that EICP has been shown to enhance the stiffness and strength of solidified soil, it has also been demonstrated to result in brittle failure of the soil, thereby impacting its erosion resistance. Researchers have found that fiber re-inforcement method (FR) can enhance the critical friction force between fibers and sand particles, thereby improving the strength of soil and preventing brittle failure. It can also provide more nucleation sites for the generation of CaCO_3_^28–30^. Therefore, in order to balance the adverse effects of brittle failure during the solidification of sand by EICP technology and improve the erosion resistance of aeolian sand, this paper combines EICP method with fiber reinforcement to solidify aeolian sand. Notably, aeolian saltation dynamics differ between hard and loose surfaces. As highlighted by Ho et al.^31^ and Kamath et al.^32^, saltation trajectories over hard crusts (e.g., EICP-solidified layers) are higher, and transport rates increase faster with shear velocity compared to loose sand. Our study focuses on soil erodibility (surface resistance), but future research should integrate these saltation dynamics to improve predictive models of sand transport in stabilized desert areas.

Compared with traditional methods, our EICP-fiber method offers unique advantages in specific scenarios. Vegetation cultivation and biogenic crusts are eco-friendly but require long-term maintenance and favorable moisture conditions, limiting their application in arid deserts with extreme climates. Fences and walls physically block sand transport but fail to improve soil properties and may disrupt local ecosystems. In contrast, EICP-fiber stabilization rapidly forms a durable surface layer (1.3–1.4 cm) with low carbon emissions, making it suitable for urgent wind erosion control in arid/semi-arid regions. Economically, it avoids the high costs of repeated vegetation planting or large-scale infrastructure, though long-term field trials are needed to compare lifecycle costs. Geomorphologically, this method is particularly indicated for stabilizing mobile dunes with loose, poorly graded sand (Cu = 2.30, Cc = 0.85), as verified in the Maowusu Desert. It has been demonstrated by preceding research that the combination of EICP and fiber reinforcement can effectively enhance the strength of solidified soil. Nevertheless, there is a paucity of research on the erosion resistance of solidified soil. This article uses EICP collaborative fiber reinforcement technology to solidify aeolian sand. By simulating different wind velocity, erosion angle, and erosion cycle in a wind tunnel testing machine, the anti-wind erosion performance of EICP, EICP-BFR, and EICP-WFR solidified aeolian sand is evaluated. Finally, considering the influence of wind velocity and erosion angle, a erosion modulus model is established and verified. The research results are significant importance in guiding wind and sand fixation projects in desert areas.

## Materials and methods

### Test materials

**Fig. 1:**
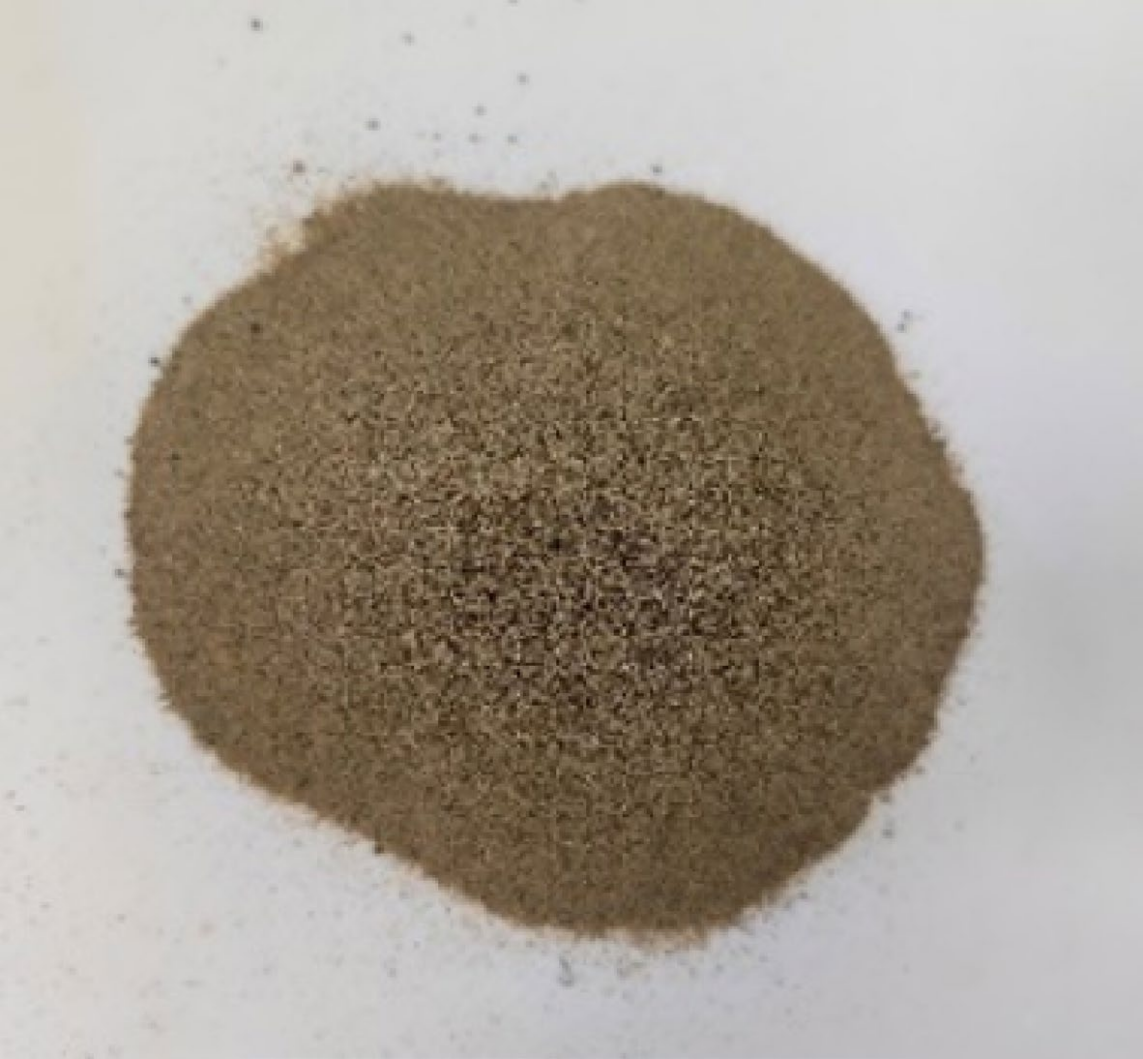
Aeolian sand.

The main material used in the experiment, aeolian sand, was taken from the Maowusu Desert in Yulin City, Shaanxi Province, as shown in Fig. 1. The sand is yellow, free of impurities, and has a uniform texture. The physical properties of aeolian sand were tested in strict accordance with the provisions set out in the “Standard for Soil Test Methods” (GB/T50123-2019)^33^, and the non-uniformity coefficient Cu and curvature coefficient Cc of aeolian sand were calculated to be 2.30 and 0.85, respectively. Therefore, aeolian sand can be classified as poorly graded fine sand. Table 1 shows the basic physical property indicators of aeolian sand used in the experiment.

**Table 1:** Basic physical property indicators of aeolian sand.

Gs	ρ (g/cm^3^)	w (%)	e	w_L_ (%)	w_*P*_ (%)	I_*P*_	C_u_	C_c_
2.65	1.59	2.60	0.67	25.00	19.60	5.40	2.30	0.85

## Urease and solidification solution

**Fig. 2:**
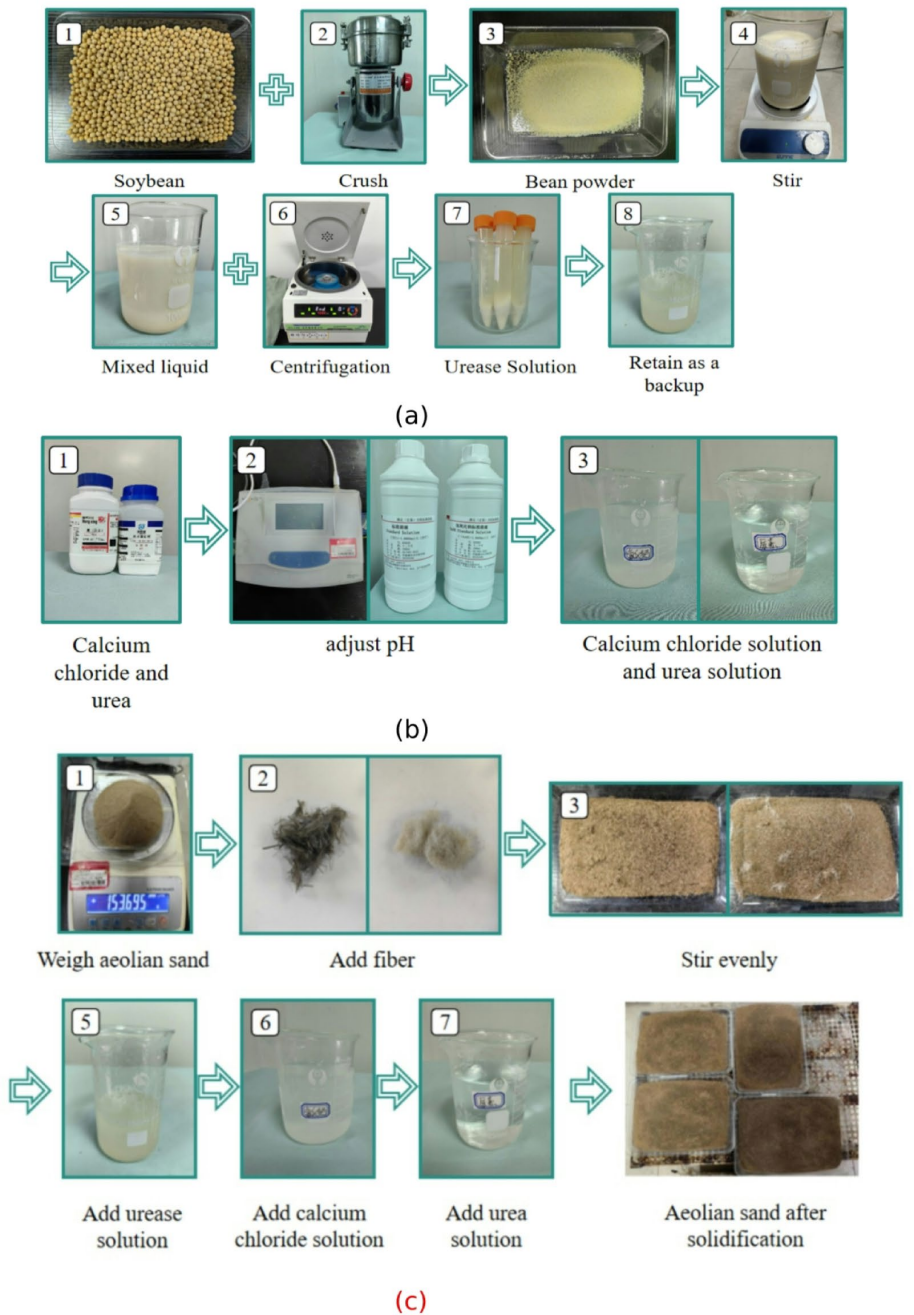
Flowchart for Preparation of Urease and Solidification Solution: (a) Urease preparation process diagram; (b) Process diagram for preparing curing solution; (c) Sand table preparation process.

The experimental soybeans are produced in Suihua City, Heilongjiang Province and purchased from the market. After drying the soybeans, grind them into soybean powder using a grinder. After sieving, add deionized water to prepare a mixture with a concentration of 100 g/L. Stir with a magnetic stirrer for 30 min to obtain the soybean liquid. Centrifuge the soybean liquid using a high-speed centrifuge at a speed of 4000 r/min for 15 min, and the supernatant taken out from the centrifuge tube is the urease solution. The curing solution consists of calcium chloride solution and urea solution. In the experiment, the calcium source of EICP was provided by anhydrous calcium chloride, with a relative molecular weight of 110.99, in the form of white cubic crystals, and easily soluble in water. Urea has a relative molecular weight of 60.06, forming transparent rod-shaped crystals that are easily soluble in water. Anhydrous calcium chloride and urea were uniformly mixed with deionized water to prepare a solution with a concentration of 1.25 mol/L. According to the “Preparation of Chemical Reagent Standard Titration Solutions” (GB/T 601–2016)^34^, HCI standard solution (c = 1.0005 mol/L) and NaOH standard solution (c = 1.0008 mol/L) were used to adjust the pH until the pH meter value showed 8.00 ± 0.05. The specific process of preparing urease and solidifying solution is shown in Fig. 2.

## Basalt fiber and wool fiber

**Fig. 3:**
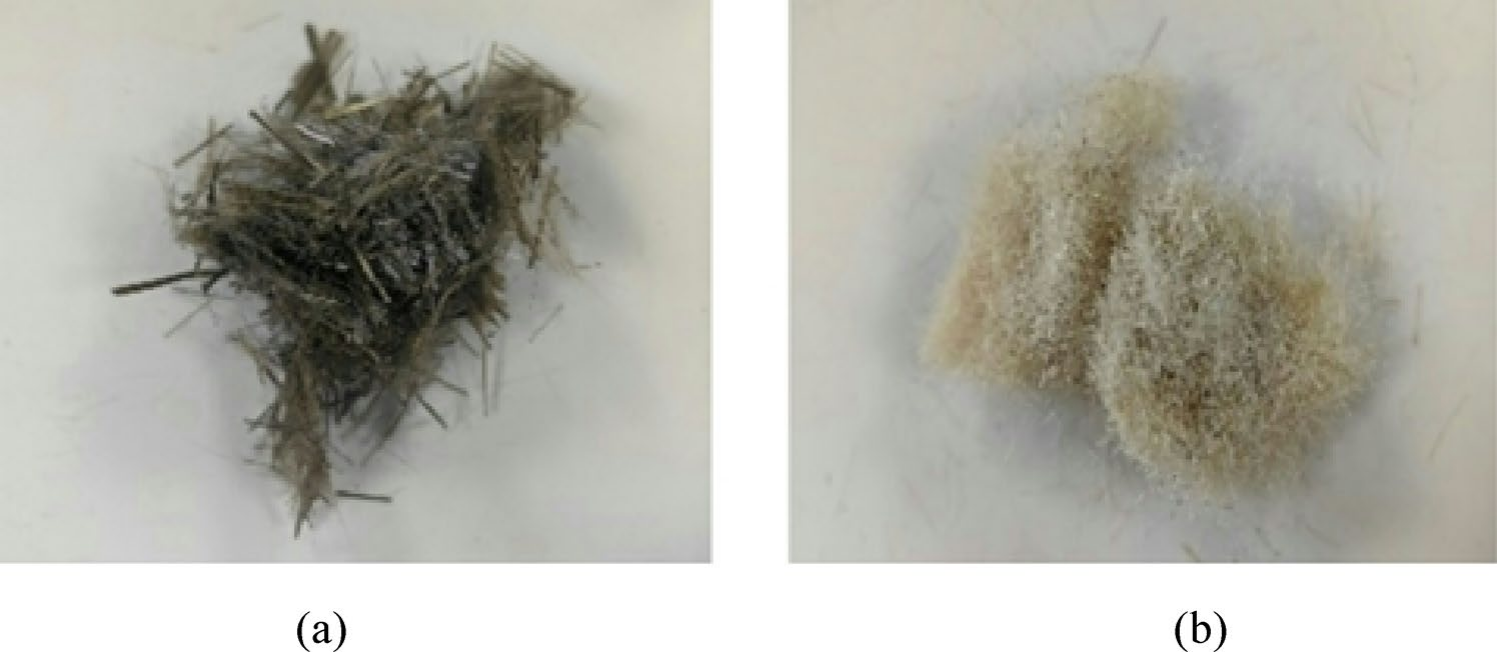
Basalt fiber and wool fiber: (a) Basalt fiber; (b) wool fiber.

Basalt fiber (BF), purchased from the market, has a smooth brown or bronze surface and is a new type of inorganic, environmentally friendly, green, high-performance fiber material. It has various excellent properties such as high strength, electrical insulation, corrosion resistance, and high temperature resistance, and is one of the four key fibers for development in China. Wool fiber (WF), purchased from the market, has a milky white color and is a natural protein fiber that often clustered and dense together, with good softness, elasticity, and wear resistance. Two types of fibers are shown in Fig. 3.

## Test equipment

**Fig. 4:**
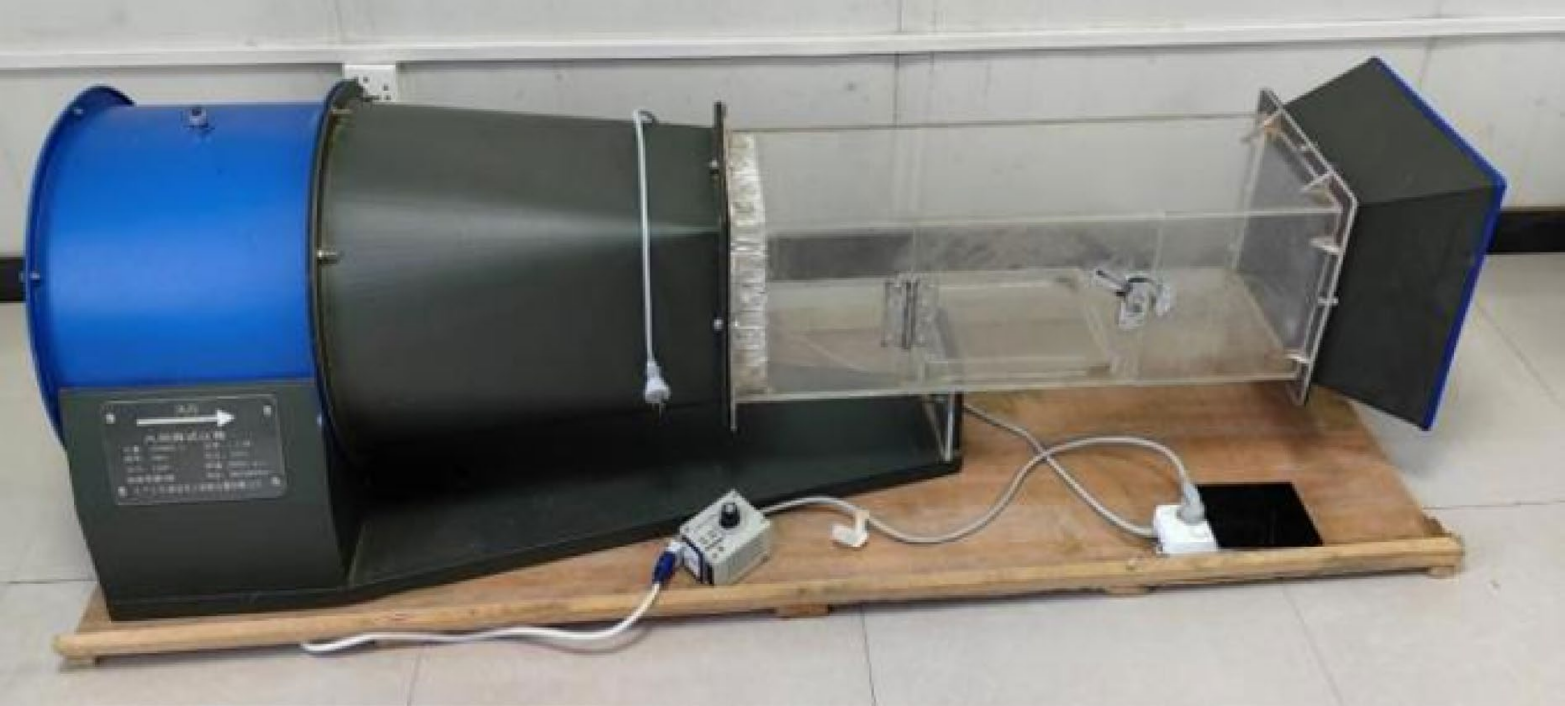
SNDY-4000 type wind tunnel testing machine.

As shown in Fig. 4, SNDY-4000 type wind tunnel test machine (Nanjing Meiwen science and Education Instrument Co., Ltd.) is used to carry out wind erosion wind tunnel model test. During the test, the HT-9829 anemometer (Shanghai Shouni Electric Technology Co., Ltd.) was used to measure the wind velocity, and the anemometer was calibrated before each test to ensure the accuracy of the speed measurement. The wind tunnel testing machine is mainly composed of three parts: the left motor part, the middle test section, and the right air outlet. Among them, the left motor is the source of wind power, with a maximum speed of 2800 r/min and a maximum air volume of 11000 m3/h. The wind direction follows a straight line from left to right in the experimental section, and the wind velocity is mainly adjusted through the connected speed regulator. The wind erosion test section in the middle is 1.0 m length, 0.3 m width, and 0.3 m height. There are iron mesh plates on both sides that connect to the motor and exhaust outlet. The left side is used to stabilize the flow, and the right side is used to slow down the blown sand. There is a groove in the middle of the bottom for placing sand table samples during the test. There is a small hole in the upper part for inserting the anemometer detection rod.

## Test method

During the preparation process of the model test sample, the aeolian sand is first sieved through a 0.5 mm sieve and placed in a 120℃ oven for 24 h before being stored for future use. The sand table mold uses a transparent plastic tray with a top opening of 23.5 cm in length and 16.5 cm in width, a bottom opening of 19.5 cm in length and 12.5 cm in width, and a height of 2.5 cm, forming an inverted trapezoid with a volume of 885.59 cm^3^ and a surface area of 387.75 cm^2^. To facilitate the discharge of urease solution and curing agent during the sample solidification process, nine evenly spaced holes with a diameter of 0.5 cm were drilled at the bottom of the small tray. To avoid sand loss, filter paper was positioned at the bottom, covering the small holes. The two-stage method is also used in the perfusion process. The solution concentration is based on the optimal curing conditions of EICP. Use a hand-held spray to evenly spray 100 mL of urease solution onto the surface of aeolian sand, wait for 1 h, and then spray 50 mL of calcium chloride solution, and finally spray 50 mL of urea solution. Repeat the above steps every 24 h for a total of 5 times, and wait for the sample to air dry naturally (about 15 days) before conducting the experiment.

The wind erosion test mainly takes wind velocity (*v*), erosion angle (*α*), and erosion cycles (*N*) as variables. During the test process, factors such as the operating power of the test equipment and the safety of laboratory electricity are considered. The wind tunnel test machine is set to blow for 5 min each time (*T*), and the test plan is shown in Table 2. The mass difference of aeolian sand in the sand table was measured before and after each experiment. Finally, a model was established and validated by analyzing the changes in wind erosion velocity and angle in the mass loss of the sand table before and after the experiment. A total of 256 sets of sand tables were made for wind erosion testing, and each set of sand table samples was set as a duplicate group.

**Table 2:** Wind erosion test scheme.

Sample	*v* (m/s)	α (°)	*N*	*T* (min)
Loose aeolian sand	3	0	1	5
EICP	6	10	2	
EICP-BFR	9	20	3	
EICP-WFR	12	30	4	

## Results and discussion

### Sample appearance analysis

**Fig. 5:**
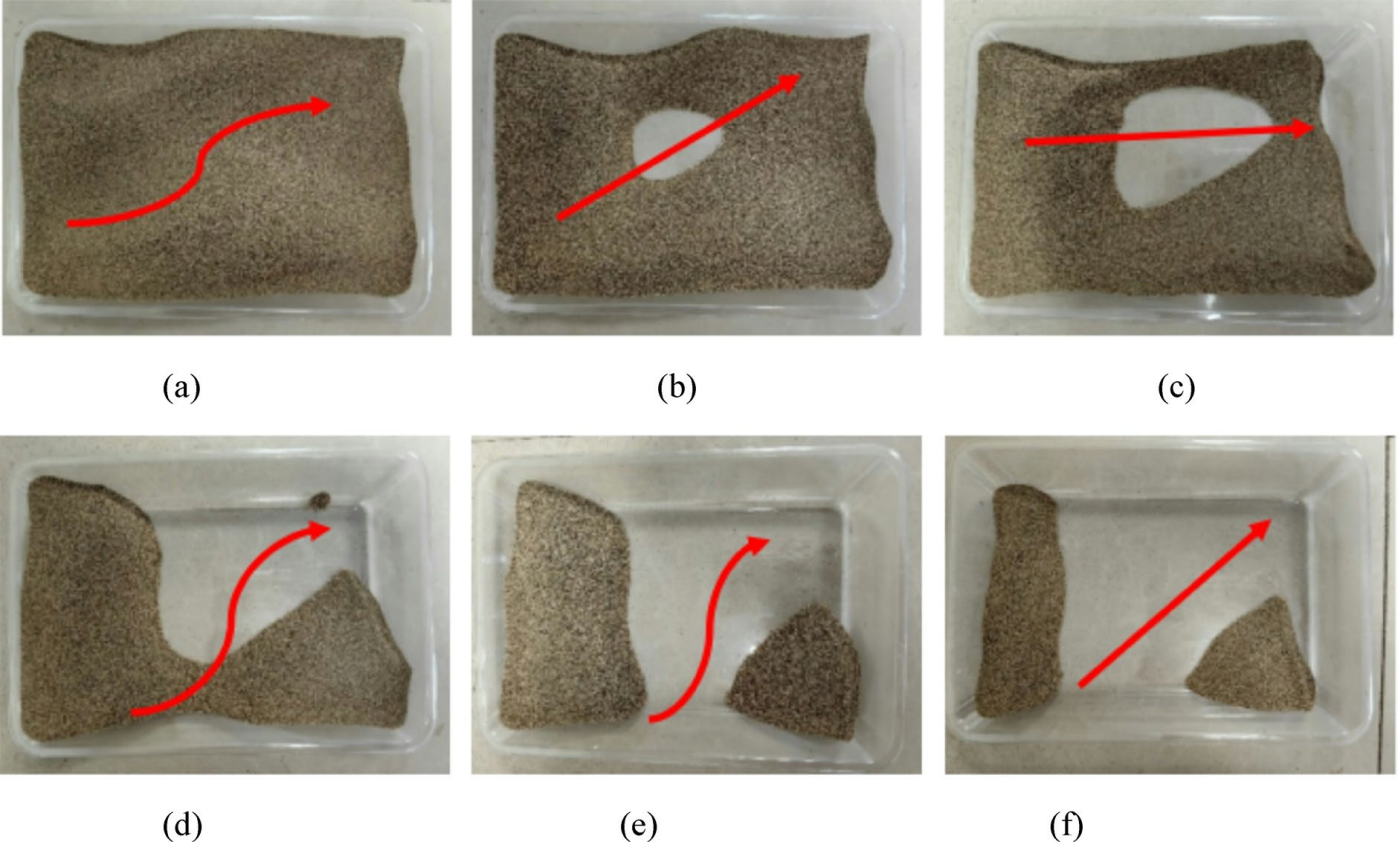
Changes in apparent state of loose sand table under wind erosion: **a** *v* = 3 m/s, α = 0°, *N* = 1; **b** *v* = 3 m/s, *α* = 10°, *N* = 2; **c** *v* = 6 m/s, *α* = 20°, *N* = 2; **d** *v* = 9 m/s, *α* = 20°, *N* = 3; **e** *v* = 9 m/s, *α* = 30°, *N* = 4; **f** *v* = 12 m/s, *α* = 30°, *N* = 4.

Figure 5 displays the wind erosion failure patterns of loose aeolian sand samples under varying speeds, angles, and cycles. The wind erosion direction is from left to right, and the arrow points to the direction in which the sand is blown away. As shown in Fig. 5a, when the wind erosion velocity (*v*) is 3 m/s, the wind erosion angle (*α*) is 0 °, and the number of wind erosion cycles (*N*) is 1, due to the small wind force and wind erosion angle, the wind action time is short, and there is relatively more accumulated sand in the sand table. The sand only flows in the middle and spreads to both sides. From Fig. 5b and c, it is evident that as the wind erosion velocity, angle, and number of wind erosion cycles increase, the first blank area in the sand table gradually increases, and the aeolian sand in the middle position is gradually blown away, causing the sand in the sand table to continuously diminish. When the wind erosion velocity and wind erosion cycle times continue to increase, as shown in Fig. 5d and e, wind erosion erodes most of the aeolian sand in the sand table, and the remaining sand in the middle changes from few to none and shows a midde fault phenomenon. Only a little aeolian sand is located close to and distant from the wind end. Finally, when the wind erosion velocity, wind erosion angle, and wind erosion cycle times reach their maximum, as shown in Fig. 5f, there is very little aeolian sand left in the sand table. It can be seen that loose aeolian sand has poor resistance to wind erosion.

**Fig. 6:**
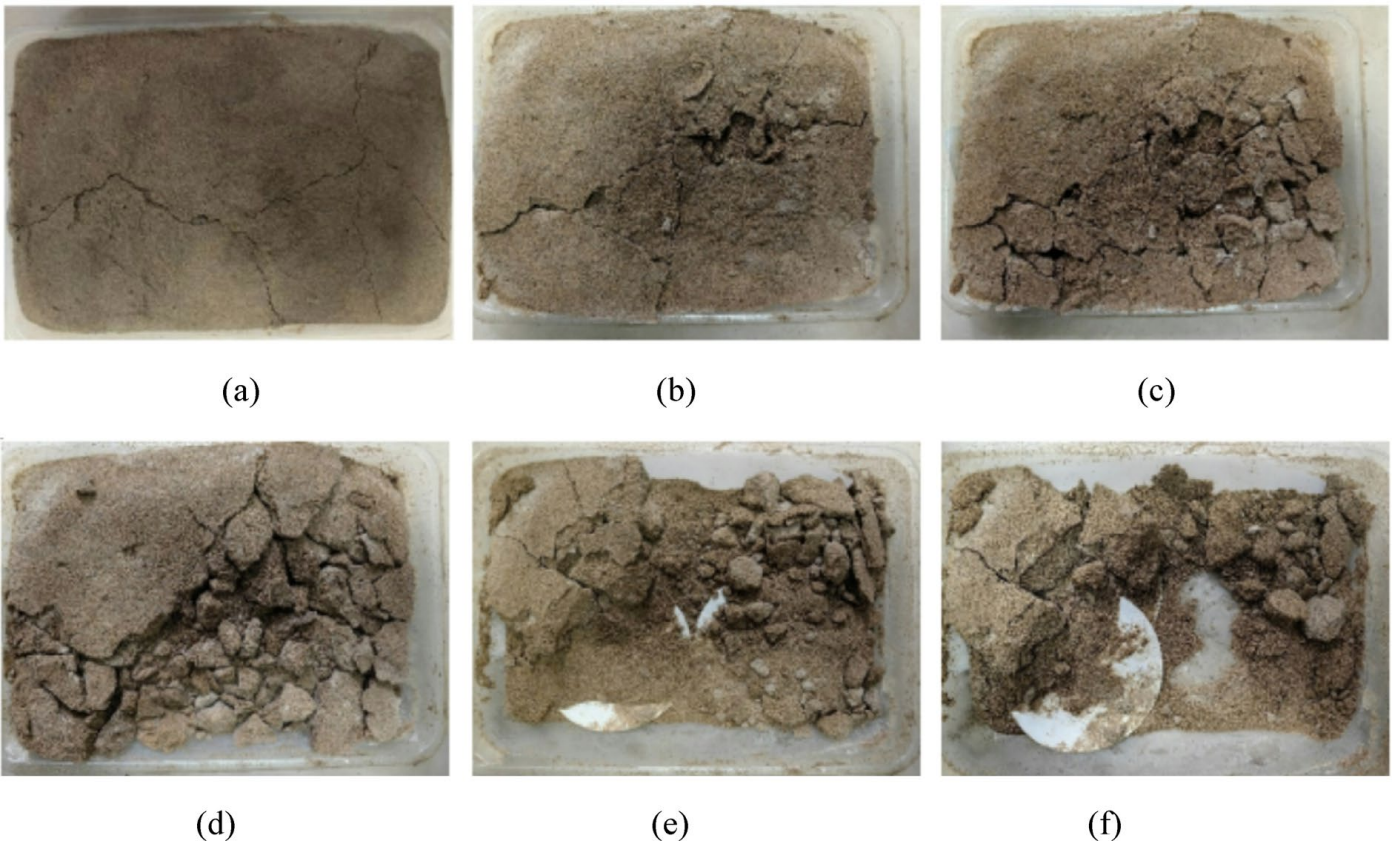
Changes in apparent state of EICP solidified aeolian sand table under wind erosion: (**a**) *v* = 3 m/s, *α* = 0°, *N* = 1; **b** *v* = 3 m/s, *α* = 10°, *N* = 2; **c** *v* = 6 m/s, *α* = 20°, *N* = 2; **d** *v* = 9 m/s, *α* = 20°, *N* = 3; **e** *v* = 9 m/s, *α* = 30°, *N* = 4; **f** *v* = 12 m/s, *α* = 30°, *N* = 4.

Figure 6 illustrates the failure patterns of EICP-solidified aeolian sand samples under varying wind erosion conditions. The direction of wind erosion is from left to right. At a wind velocity of 3 m/s, an angle of 0°, and 1 cycle, the erosion amount of the solidified sand table is much smaller compared to loose aeolian sand, and the surface hard shell effectively prevents erosion. The surface floating sand on the sand table is removed by wind, and cracks appear in the thin solidified layer. When the wind velocity remains constant at 10° angle and 2 cycles, cracks in the sand table spread, the surface loose sand was blown away, and a small amount of CaCO_3_ was exposed. As the wind erosion conditions increase, the sand changes from large blocks to small pieces, and the sharp surface becomes rounded. At a wind velocity of 12 m/s and a 30° angle, the wind erosion force and area expand, the solidified layer is blown away, and the unconsolidated loose sand is blown away, leaving only the deeply consolidated parts and the bottom layer of sand. The experiment found that CaCO_3_ crystals mainly precipitate in shallow layers, as EICP only solidifies the surface layer of aeolian sand, which is consistent with the experimental results of Wang et al.^35^. This indicates that EICP solidified aeolian sand can form a hard surface and possesses some capability to withstand wind erosion.

**Fig. 7:**
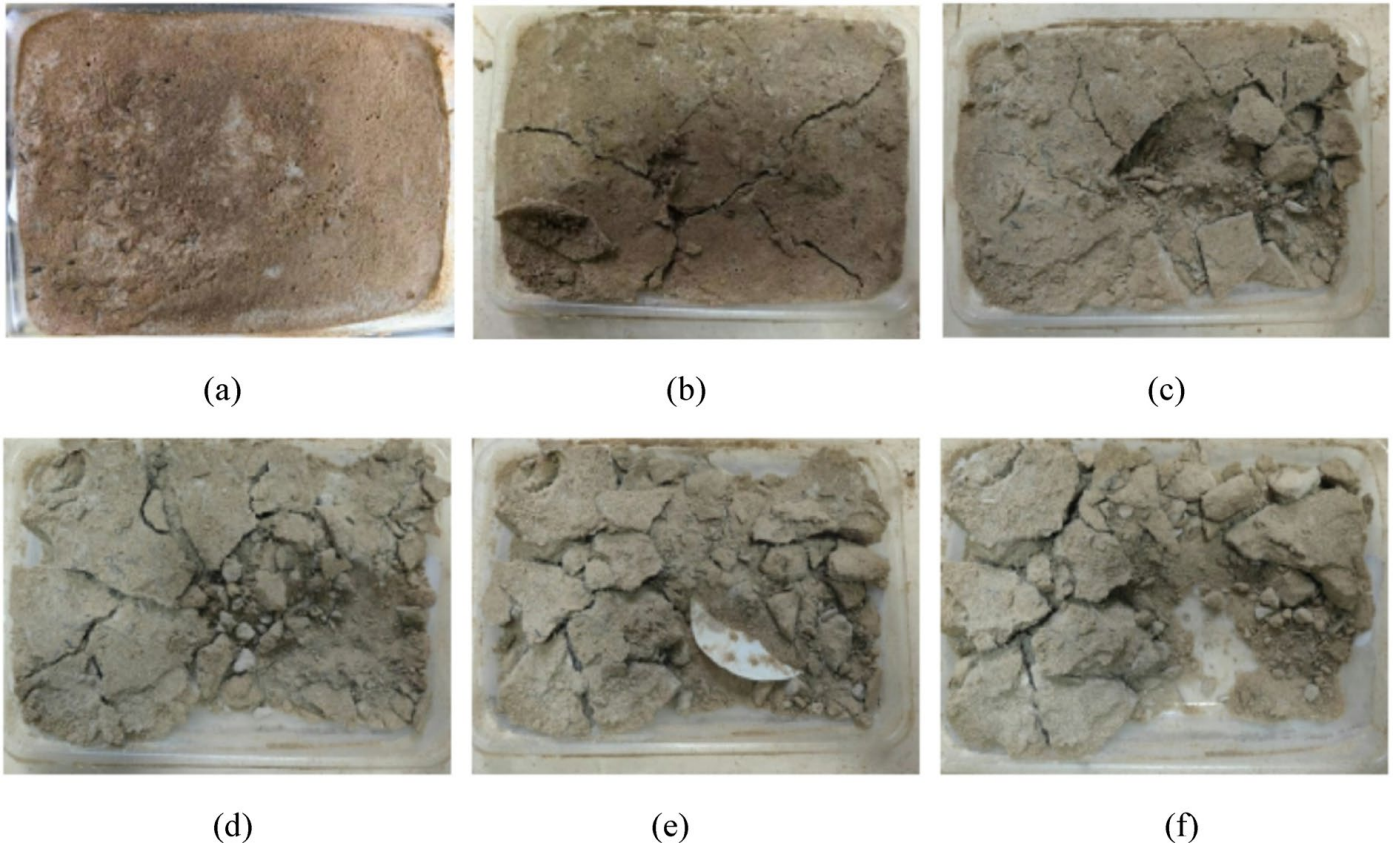
Changes in apparent state of EICP-BFR solidified aeolian sand table under wind erosion: (a) *v* = 3 m/s, *α* = 0°, *N* = 1; **b** *v* = 3 m/s, *α* = 10°, *N* = 2; **c** *v* = 6 m/s, *α* = 20°, *N* = 2; **d** *v* = 9 m/s, *α* = 20°, *N* = 3; **e** *v* = 9 m/s, *α* = 30°, *N* = 4; **f** *v* = 12 m/s, *α* = 30°, *N* = 4.

**Fig. 8:**
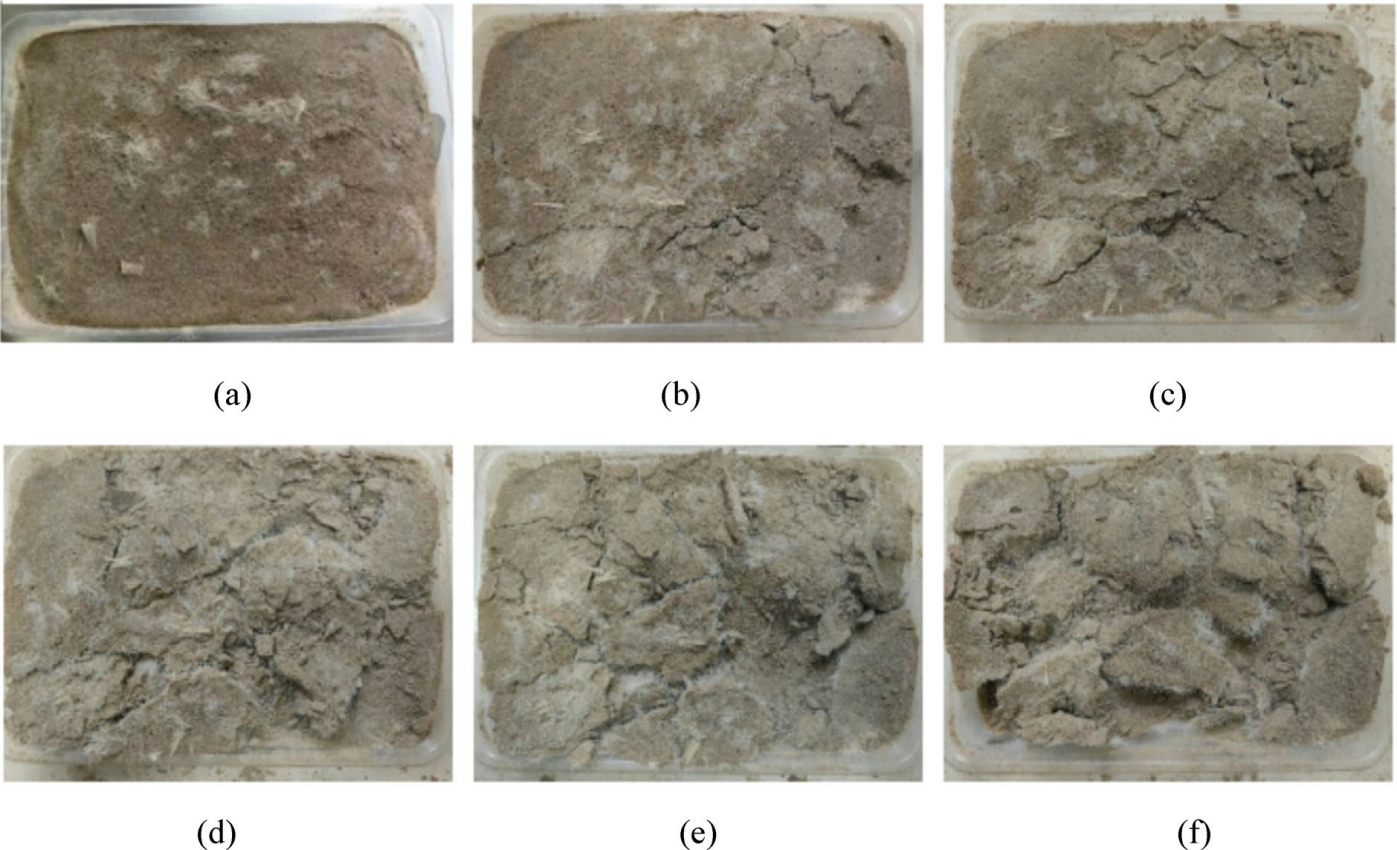
Changes in apparent state of EICP-WFR solidified aeolian sand table under wind erosion: **a** *v* = 3 m/s, *α* = 0°, *N* = 1; **b** *v* = 3 m/s, *α* = 10°, *N* = 2; **c ***v* = 6 m/s, *α* = 20°, *N* = 2; **d ***v* = 9 m/s, *α* = 20°, *N* = 3; **e** *v* = 9 m/s, *α* = 30°, *N* = 4; **f** *v* = 12 m/s, *α* = 30°, *N* = 4.

Figures 7 and 8 show the failure modes of EICP-BFR and EICP-WFR solidified aeolian sand specimens under different wind erosion conditions. The results showed that under the minimum wind erosion conditions, only slight damage occurred on the surface of the aeolian sand table with added fibers. As the wind erosion velocity and angle increase, the EICP-BFR sand table cracks and gradually fractures, while the EICP-WFR sand table exhibits better resistance to wind erosion with fewer cracks, and wool fibers help protect uncured aeolian sand. In extreme wind erosion scenarios, the middle section of the EICP-BFR sand table incurs greater losses, while the EICP-WFR sand table suffers fewer losses due to the “pulling” effect of fibers, protecting the bottom layer of aeolian sand. It has been demonstrated that the wind erosion loss of the EICP fiber-reinforced solidified sand table is significantly lower than that of the sand table solidified only by EICP, indicating that fiber reinforcement can significantly enhance the sample’s strength and wind erosion resistance.

## Analysis of the impact of wind velocity

Wind erosion velocity plays a key role in determining the threshold friction velocity (TFV), flight altitude, and transport distance of aeolian sand. After experiencing wind erosion, there were significant differences in the mass difference of aeolian sand in the sand table before and after the experiment among the four samples. The mass loss rates of aeolian sand, represented by *η*, as shown in Eq. (1)

1 $$\eta =\frac{{{m_0} - m}}{{{m_0}}}$$

In which, *η* - mass loss rate, %; *m* - mass of aeolian sand in the sand table after wind erosion, g; *m*_0_- mass of aeolian sand in the sand table before wind erosion, g.

**Fig. 9:**
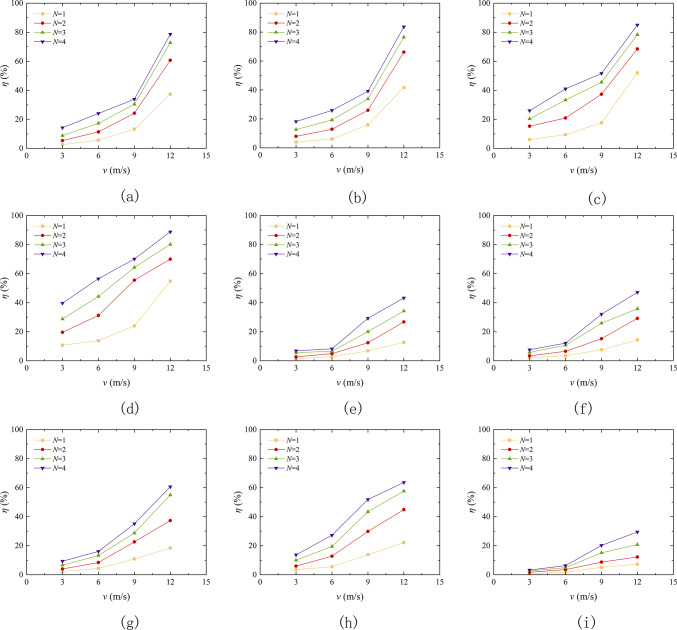

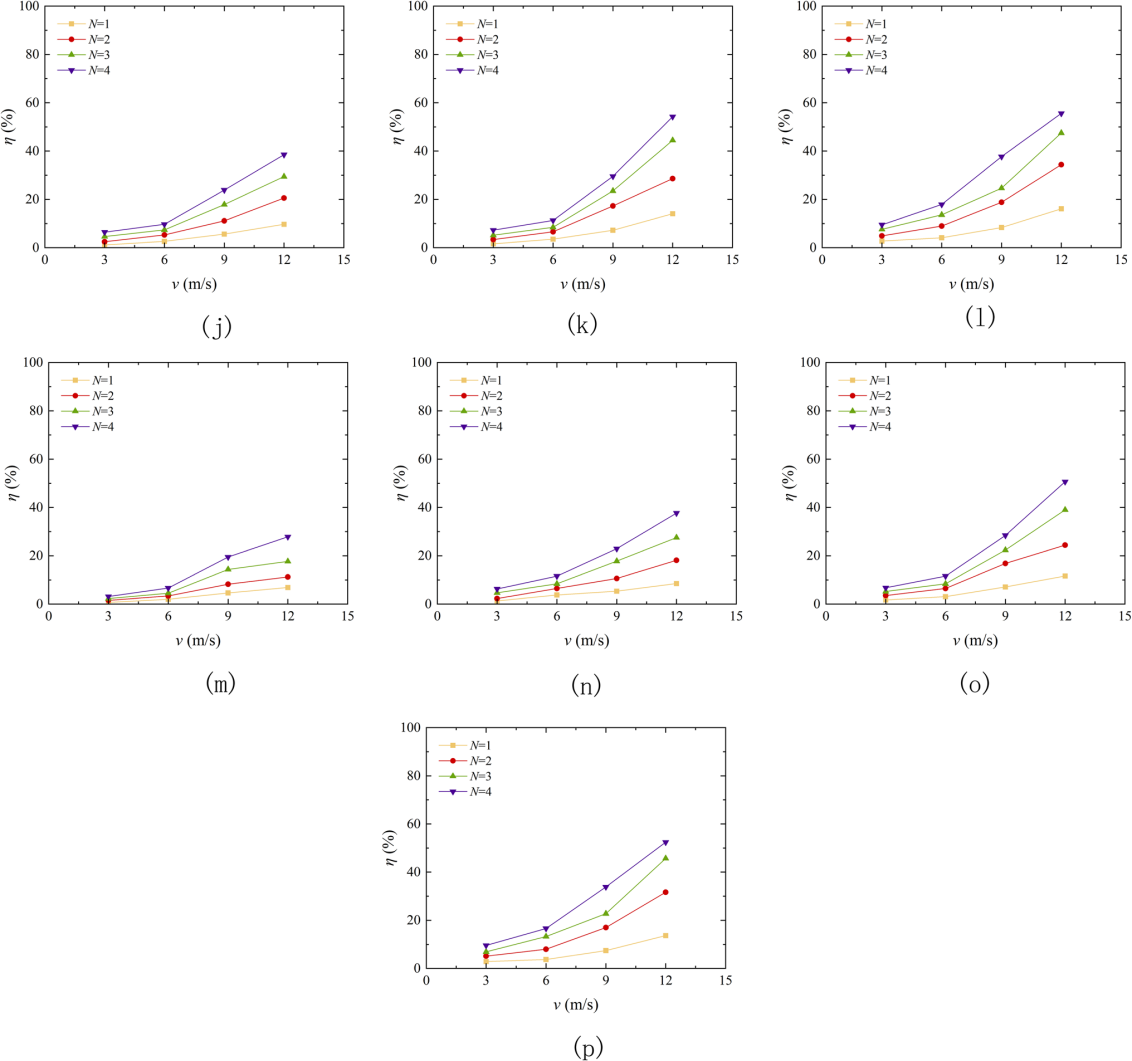
Curve of mass loss rate variation of aeolian sand table with wind erosion velocity: **a** Aeolian sand, *α* = 0°; **b** Aeolian sand, *α* = 10°; **c** Aeolian sand, *α* = 20°; **d** Aeolian sand, *α* = 30°; **e** EICP, *α* = 0°; **f** EICP, *α* = 10°; **g** EICP, *α* = 20°; **h** EICP, *α* = 30°; **i** EICP-BFR, *α* = 0°; **j** EICP-BFR, *α* = 10°; **k** EICP-BFR, *α* = 20°; **k** EICP-BFR, *α* = 30°; **m**EICP-WFR, *α* = 0°; **n** EICP-WFR, *α* = 10°; **o** EICP-WFR, *α* = 20°; **p** EICP-WFR, *α* = 30°.

In general, the smaller the *η*, the more accumulated sand in the sand table, indicates better resistance to wind erosion of the sample. Figure 9 shows the variation curves of mass loss rate of different types of aeolian sand tables with wind velocity under different erosion cycles and erosion angles. As demonstrated in the figure, there is a direct correlation between the rate of mass loss of aeolian sand and the velocity of wind erosion. Nikseresht et al.^36^ also conducted wind tunnel model tests and obtained similar conclusions. When the wind erosion velocity increases from 3 m/s to 12 m/s, the mass loss rate range of loose sand table is 2.36% -88.79%, the mass loss rate range of EICP solidified aeolian sand table is 1.46% -63.55%, the mass loss rate range of EICP-BFR solidified aeolian sand table is 0.82% -55.57%, and the mass loss rate range of EICP-WFR solidified aeolian sand table is 0.71% -52.40%. As shown in Fig. 9e–p, with the continuous increase of wind velocity, the growth rate of mass loss rate of aeolian sand also increases. The main reason for this situation is that a certain thickness of solidified layer is formed on the surface of the sand table after solidification, and the solid surface of the solidified aeolian sand cannot be destroyed under low wind velocity^37^, thereby increasing TFV. Generally, the higher the TFV, the stronger the sample resistance to wind erosion^38^. When the wind velocity reaches the critical point of the sample, the bonding layer on the surface of the sand table is destroyed, so the mass loss rate of aeolian sand in the sand table will rapidly increase. From the experimental results, it is evident that the ability of loose sand to resist wind erosion is limit. After EICP solidification, it can effectively reduce the mass loss rate in the sand table. This provides reference significance for the application of EICP solidification of aeolian sand in wind and sand fixation engineering. On the basis of EICP, the combination of fiber reinforcement technology to solidify aeolian sand greatly reduces the loss rate of aeolian sand, which proves the feasibility of EICP combined with fiber reinforcement to solidify aeolian sand.

### Analysis of the impact of erosion angle

**Fig. 10:**
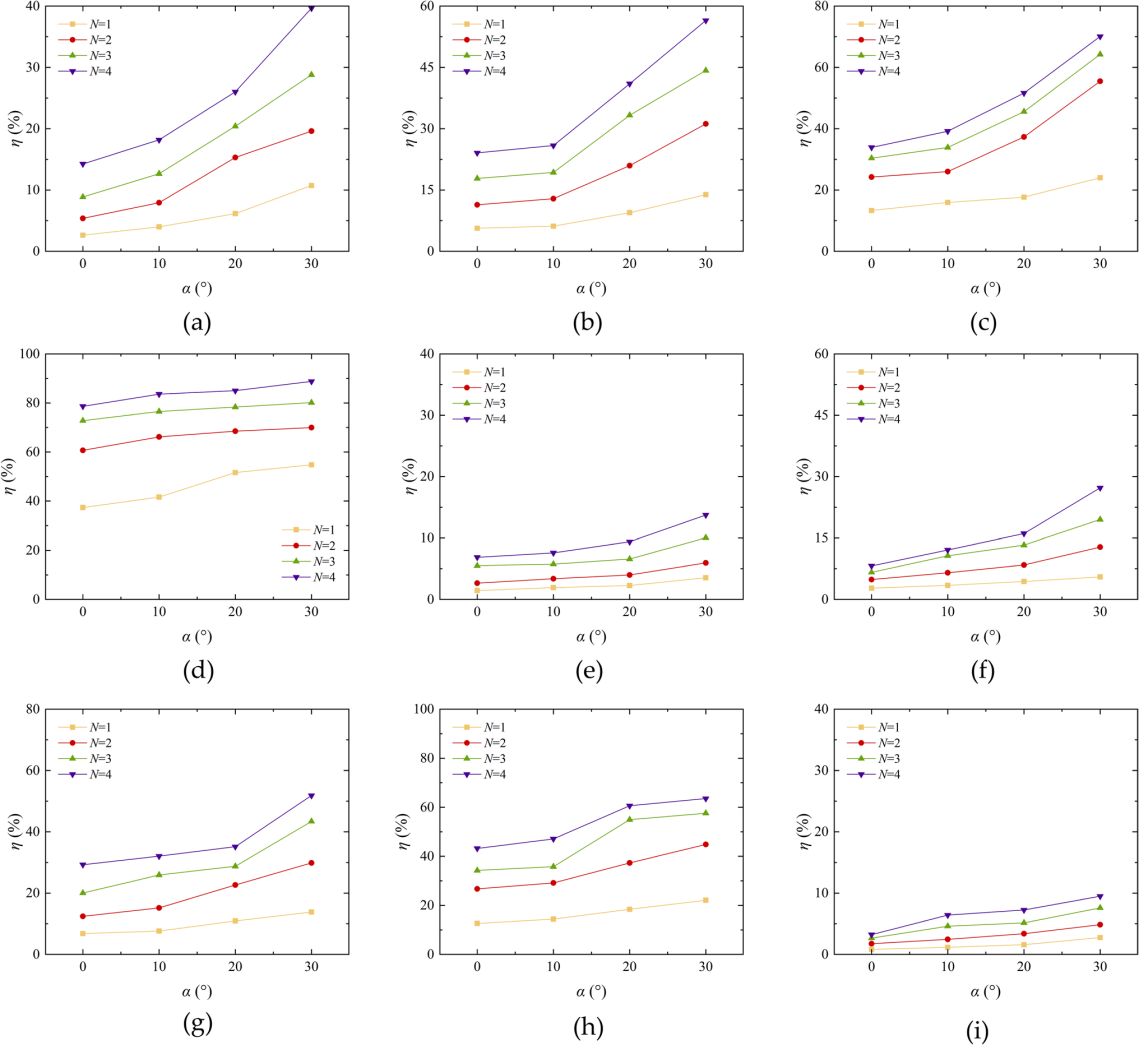

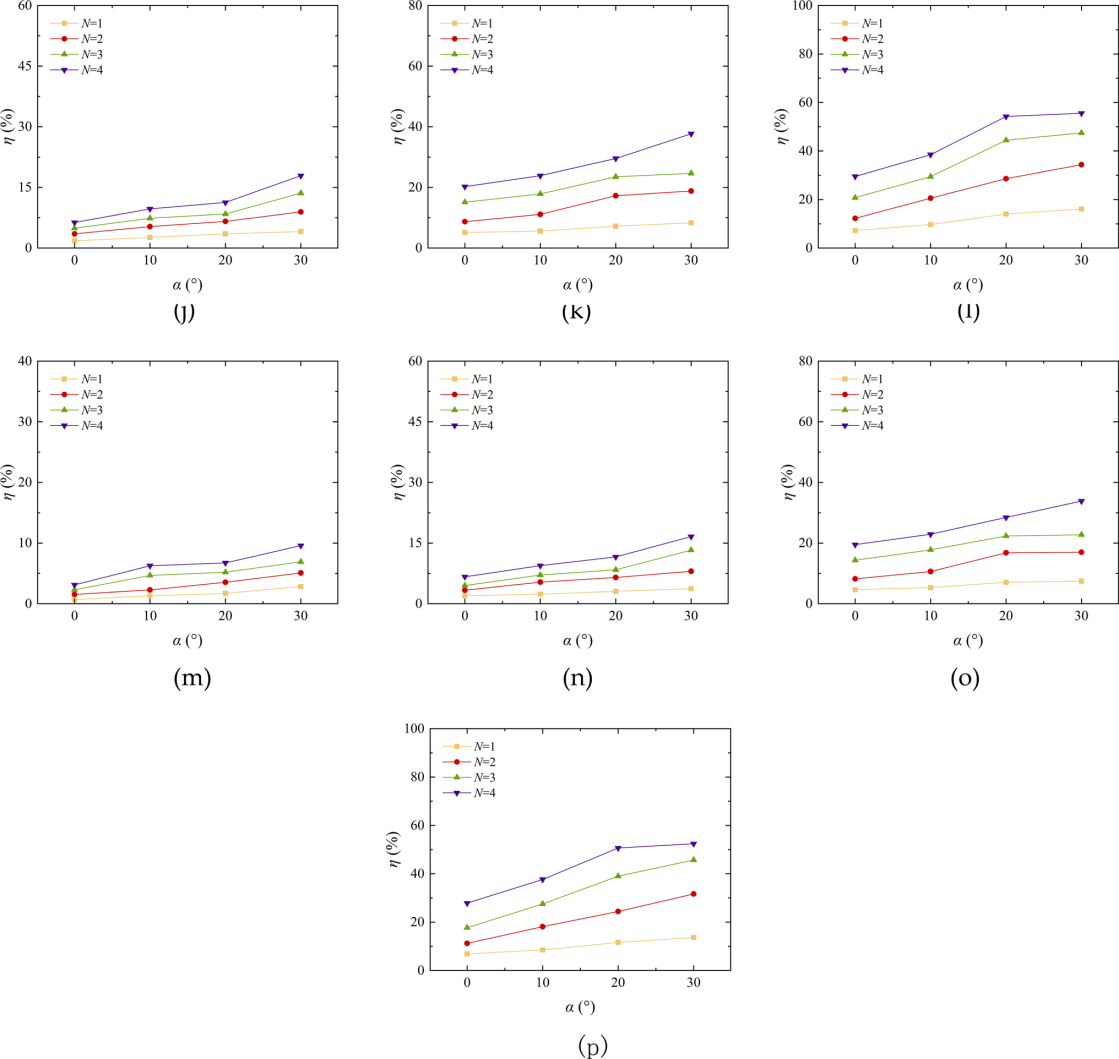
Quality loss rate variation curve of aeolian sand table with wind erosion angle: **a** Aeolian sand, v = 3 m/s; **b** Aeolian sand, v = 6 m/s; **c** Aeolian sand, v = 9 m/s; **d** Aeolian sand, *v* = 12 m/s; **e** EICP, *v* = 3 m/s; **f** EICP, *v* = 6 m/s; **g** EICP, *v* = 9 m/s; **h** EICP, *v* = 12 m/s; **i** EICP-BFR, *v* = 3 m/s; **j** EICP-BFR, *v* = 6 m/s; **k** EICP-BFR, *v* = 9 m/s; **l** EICP-BFR, *v* = 12 m/s; **m**EICP-WFR, *v* = 3 m/s; **n** EICP-WFR, *v* = 6 m/s; **o** EICP-WFR, *v* = 9 m/s; **p** EICP-WFR, *v* = 12 m/s.

The angle of wind erosion exerts a substantial influence on the rate of quality loss of aeolian sand. Figure 10 shows the curve of the mass loss rate of aeolian sand under different erosion angle. The findings revealed that as the erosion angle increased from 0° to 30°, the mass loss rate increased. Tominaga et al.^39^ conducted wind erosion wind tunnel tests on a 200 mm wide cube model standing on a sand surface, and found that a greater degree of erosion occurred at the windward angle of the cube than in the surrounding area. This conclusion is consistent with the conclusion of this section. Taking EICP solidified aeolian sand as an example, with a wind erosion velocity of 3 m/s and 4 cycles, the mass loss rate increased from 6.87 to 13.73%, showing a stepwise increase. The wind angle changes smoothly from 0° to 10°, while it changes significantly from 10° to 30°, indicating that an increase in wind erosion angle leads to an increase in the contact area between the sand table surface and the wind, an enhanced wind erosion effect, a decrease in TFV, and easy dispersion of sand particles. The solidified sand table is more resistant to wind erosion than loose sand, and the difference in wind erosion resistance between EICP-BFR and EICP-WFR solidified sand tables is not significant. The mass loss rate of loose aeolian sand increases approximately horizontally when the erosion angle between 20° and 30°, while the solidified sand increases with the increase of erosion angle under other conditions, indicating that EICP solidification significantly improves the wind erosion resistance of aeolian sand.

### Analysis of the influence of erosion cycles

**Fig. 11:**
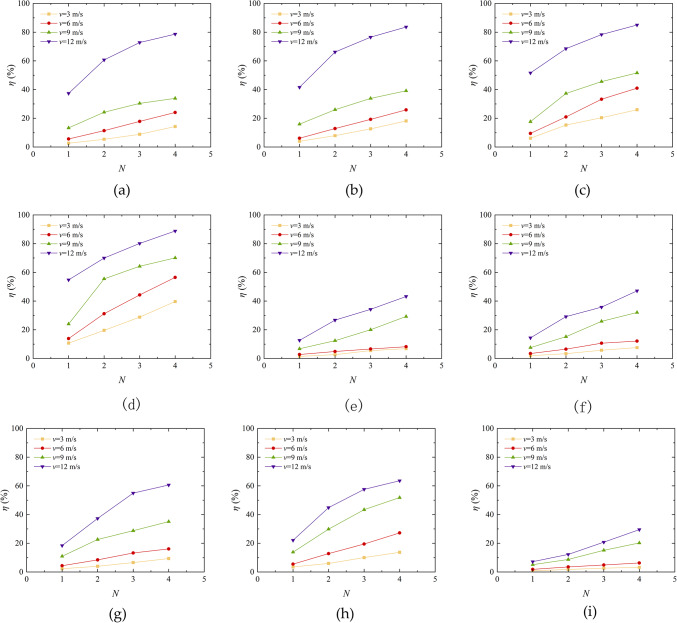

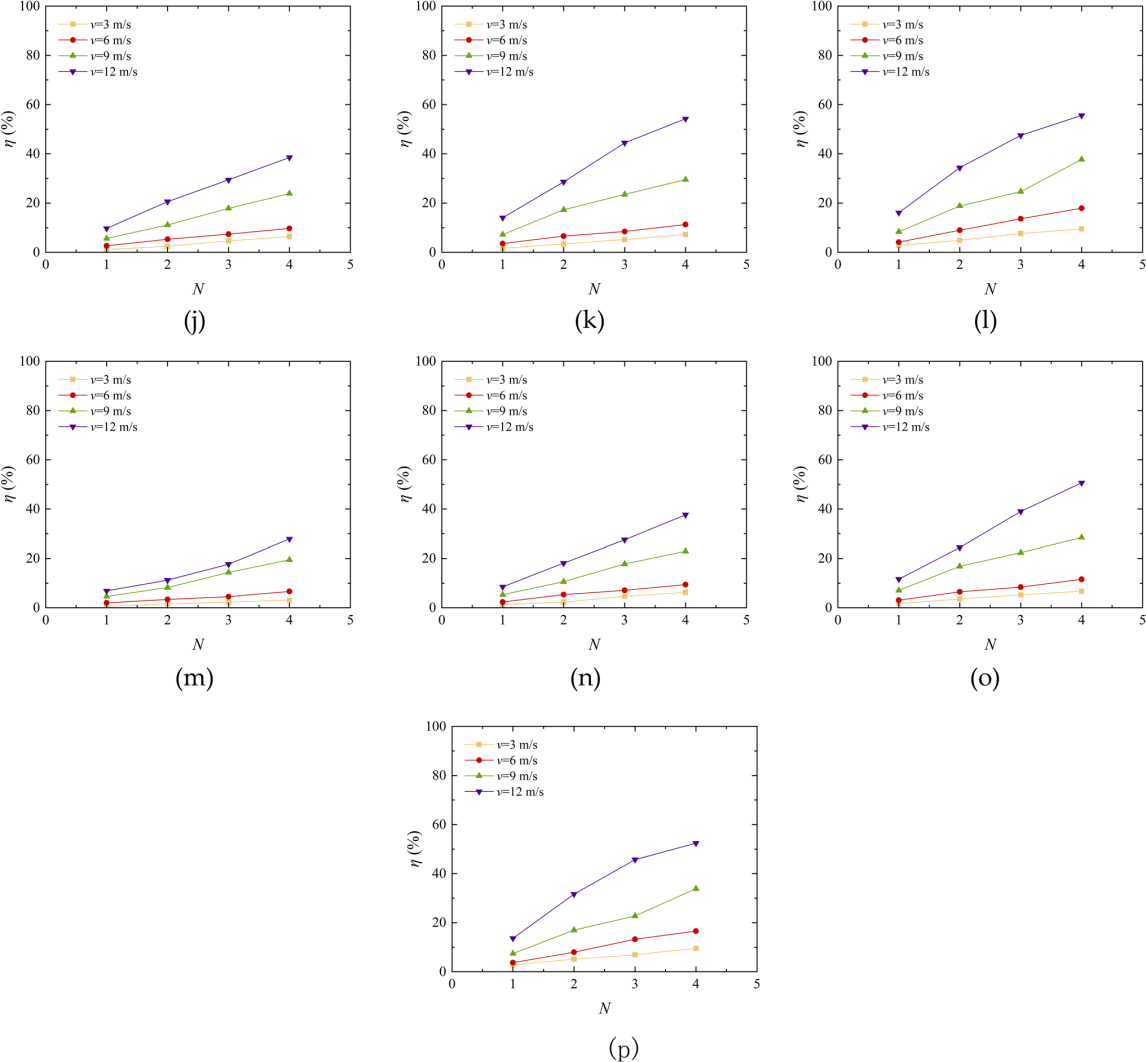
Quality loss rate variation curve of aeolian sand table with wind erosion cycles: **a** Aeolian sand, *α* = 0°; **b** Aeolian sand, *α* = 10°; **c** Aeolian sand, *α* = 20°; **d** Aeolian sand, *α* = 30°; **e** EICP, *α* = 0°; **f** EICP, *α* = 10°; **g** EICP, *α* = 20°; **h** EICP, *α* = 30°; **i** ICP-BFR, *α* = 0°; **j** EICP-BFR, *α* = 10°; **k** EICP-BFR, *α* = 20°; **l**E ICP-BFR, *α* = 30°; **m** ICP-WFR, *α* = 0°; **n** EICP-WFR, *α* = 10°; **o** EICP-WFR, *α* = 20°; **p** EICP-WFR, *α* = 30°.

The number of erosion cycles is a key factor affecting the quality loss of aeolian sand. In harsh desert environments, the ability of solidified aeolian sand to resist wind erosion in the long term is the main challenge. As illustrated in Fig. 11, the rate of mass loss exhibited by different types of aeolian sand is shown to vary with increasing wind erosion frequency, as well as with varying wind erosion velocity and angles. The results show that an increase in wind erosion frequency leads to an increase in quality loss rate, especially when the wind erosion angle is large. The quality loss rates of EICP, EICP-BFR, and EICP-WFR solidified aeolian sand increased from 1.46%, 0.82%, and 0.71–7.59%, 6.41%, and 6.26%, respectively, it is evident that the incorporation of fiber reinforcement has led to a substantial enhancement in the wind erosion resistance of aeolian sand. This is consistent with the conclusion of reference^40^. The primary cause of this phenomenon is that with the number of erosion cycles is small, the wind erosion time is short, and the surface of aeolian sand is less affected by wind erosion. The hardened layer of aeolian sand remains partially intact, contributing to a lower rate of mass loss^41^. When the frequency of wind erosion is low, the surface erosion of aeolian sand is minimal and the rate of quality loss is low. As the frequency increases, the erosion time prolongs, the solidified layer of aeolian sand is destroyed, the loose sand inside is blown away, and the quality loss rate increases. The increase in wind erosion frequency is equivalent to the increase in total wind erosion time, and the aeolian sand reinforced with EICP and fiber reinforcement is more resistant to wind erosion than EICP solidification and loose sand.

### Analysis of wind erosion mechanism

**Fig. 12:**
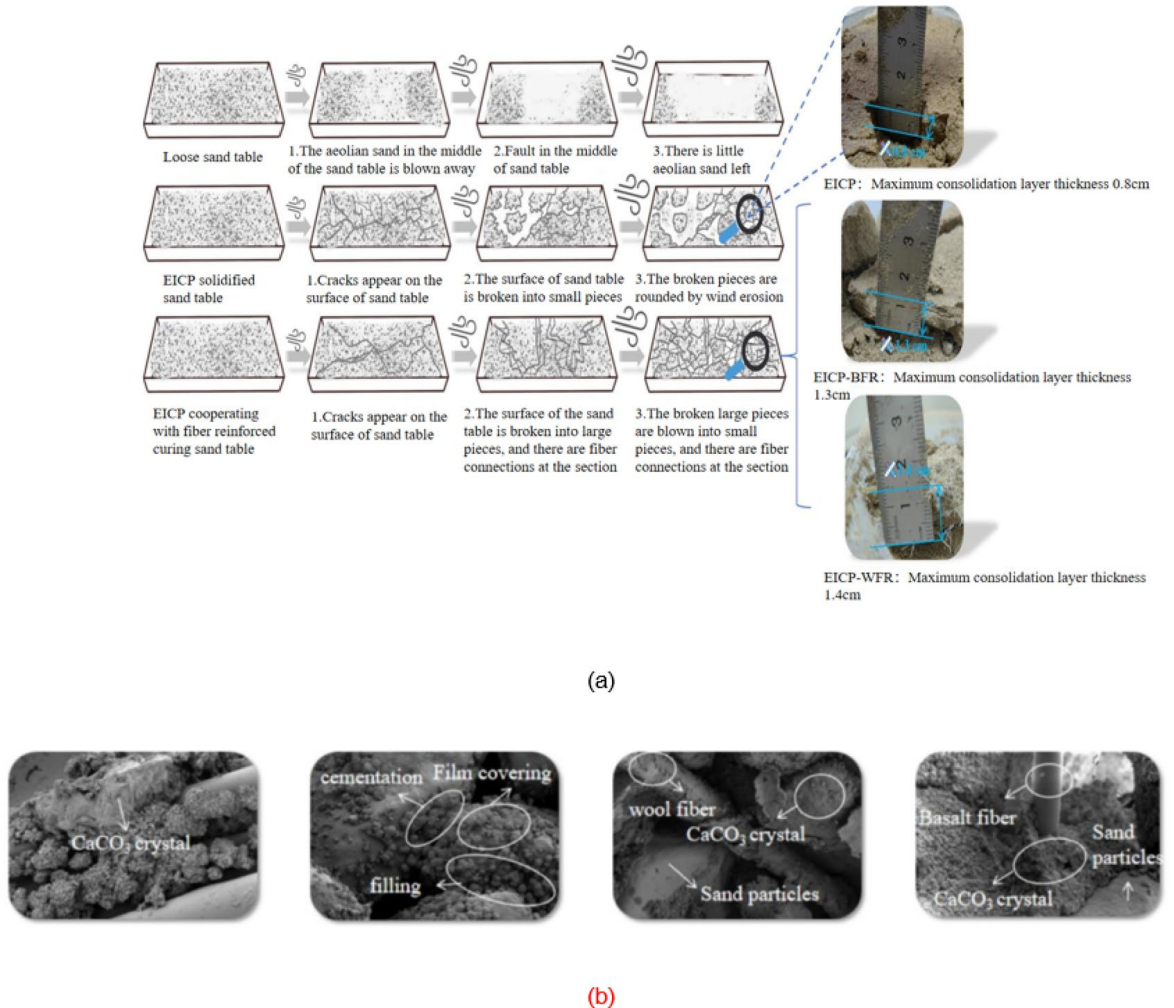
Mechanism diagram under wind erosion of aeolian sand: **a** Schematic diagram of wind erosion process of aeolian sand; **b** Mechanism of action of EICP and EICP-FR in solidifying wind-blown sand.

Figure 12 shows the mechanism of wind erosion process of aeolian sand. The aeolian sand particles themselves are loose. When the loose sand is eroded by the wind, the sand in the middle is gradually blown away, then faults appear, and finally there is little sand left in the sand table. When the wind force blows and transports the sand particles on the surface of the sand table solidified by EICP, the original single sand particles are solidified into a whole to increase their own weight, which greatly reduces the phenomenon of “wind blowing” of sand particles on the surface of the sand table, thus significantly reducing the quality loss of aeolian sand on the table. The solidified layer on the surface of the sand table is blown apart at first, then the crack develops and the solidified layer is broken into large pieces, and finally the fracture surface between the large pieces is abraded and broken into small pieces. When fibers are added on the basis of EICP solidification, the fibers mainly play two roles: first, they are staggered between the pores of sand particles, providing more nucleation sites for the formation of CaCO_3_ crystals, improving the solidification strength of the hard shell layer on the surface of the sand table, and increasing the wind erosion starting velocity of sand particles; Second, “bridge” adjacent sand particles, enhance the bonding strength between particles, bear some external forces, and form aggregates with certain strength and stiffness on the surface of the sand table. When the wind erosion conditions increase, the aeolian sand solidified by EICP-BFR and EICP-WFR relies on the pulling effect of fibers, which reduces the separation and abrasion of small groups of sand particles and small blocks. At this time, the interaction between sand particles, CaCO_3_ crystals and fibers can overcome the lifting force and impact force brought by part of the air flow, so the mass loss of aeolian sand solidified by EICP-BFR and EICP-WFR is less than that solidified by EICP.

In addition, it is also found that the consolidation layer thickness of EICP, EICP-BFR and EICP-WFR solidified aeolian sand table is uneven, which is also one of the reasons for the different mass loss rate of aeolian sand quality. The measurement shows that the maximum thickness of consolidation layer of aeolian sand solidified by EICP is 0.8 cm, and the maximum thickness of consolidation layer of aeolian sand solidified by EICP-BFR and EICP-WFR are 1.3 cm and 1.4 cm respectively. In the process of wind erosion, it is also found that, compared with loose sand, the solidified sand surface first appears obvious cracks under the effect of wind erosion, and the sand particles in the cracks are protected under the wind erosion without being blown away. The main reason is that the formation of a solid layer restricts the movement of sand particles. The thickness of consolidation layer increases after adding fiber, which proves that the addition of fiber can effectively promote the cementation of CaCO_3_ crystal, improve the surface strength and wind erosion resistance, and reduce the quality loss of aeolian sand.

EICP technology solidifies loose wind-deposited sand into a cohesive mass primarily through the solidification effect of calcium carbonate. As shown in the figure, CaCO_2_ crystals are primarily distributed between sand particles, within the pores of sand particles, and on the surfaces of sand particles, thereby serving to bind, fill, and coat the sand particles. CaCO_2_ crystals deposit, aggregate, and grow between adjacent sand particles, bonding them into a cohesive unit. This transforms the contact between particles from point contact to surface contact, thereby enhancing the overall stability of the sample. The coverage of CaCO_2_ crystals on the surfaces of sand particles acts as a coating^22^. The bonding and filling effects of CaCO_2_ crystals are the primary mechanisms for reinforcing wind-deposited sand, while the reinforcing effect of the coating^42–44^ is not significant. However, the coating effect increases the roughness and interlocking force of sand particles. During deformation under load, the friction between CaCO_2_ crystals and sand particles hinders deformation, thereby enhancing the mechanical strength of the sample.

#### Erosion modulus model establishment and validation

##### Model establishment

Due to the limit erosion resistance of loose aeolian sand, when the erosion velocity, angle, and number of wind erosion cycles continue to increase, the *η* of loose aeolian sand approaches horizontal growth, mainly because the quality loss of loose aeolian sand quickly reaches its maximum value. The experiment also found that there was not much difference in quality loss between the aeolian sand cured by EICP-BFR and the aeolian sand cured by EICP-WFR. Therefore, an erosion modulus model for the aeolian sand solidified by EICP and EICP-WFR under wind erosion was established and verified. In order to better evaluate the ability of aeolian sand to resist wind erosion, the wind erosion modulus is introduced to evaluate the erosion resistance by considering the influence of wind erosion time^45]– [46^. The expression for wind erosion modulus is:

2 $$E=\frac{{{m_0} - m}}{{TNA}}$$

In which, *E* represents the wind erosion modulus, g/(m^2^·min);*m* - mass of aeolian sand in the sand table after wind erosion, g; *m*_0_- mass of aeolian sand in the sand table before wind erosion, g; *T* - duration of each erosion, min༛*N* - number of wind erosion cycles; *A* - surface area of aeolian sand table.

To analyze the variation of wind erosion modulus of aeolian sand with wind erosion velocity and angle, the assumed expression is:

3 $$E=f\left( {v,a} \right)$$

In which, *E* represents the wind erosion modulus of the solidified aeolian sand table, g/(m^2^·min); *v* - wind erosion rate, m/s; *α* - Wind erosion angle, °.

The analysis of experimental results indicates that the growth trend of parameter *η* is significant when the erosion angle is between 0° and 20°, and slows down when the erosion angle is between 20° and 30°. Therefore, we attempted to use piecewise functions to establish relationship models between different wind erosion angles, respectively. The expression is:

4 $$E=\left\{ \begin{gathered} y{v^x} \hfill \\ {x_1}v+c \hfill \\ \end{gathered} \right.\quad \quad \begin{array}{*{20}{c}} {\alpha \leqslant 20^\circ } \\ {\alpha >30^\circ } \end{array}$$

In which, *x*, *y*, and *x’* are model parameters, and *c* is a constant, which can be obtained through regression analysis.

By analyzing *x* and *y* with the wind erosion angle *α*, it can be seen that *x* and *y* increase linearly with the increase of wind erosion angle *α*. Equation (5) can be expressed as:

In which, *x*_1_ and *y*_1_ are model parameters, and *c*_1_ and *c*_2_ are constants, which can be obtained through regression analysis.

#### Parameter acquisition

**Fig. 13:**
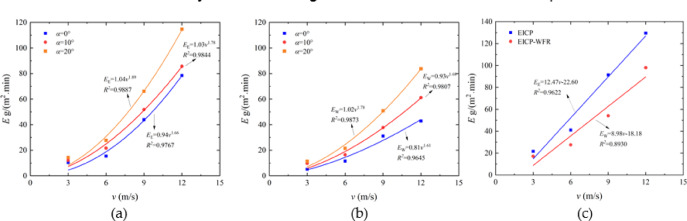
Erosion modulus model establishment for solidified aeolian sand: **a** EICP, *α* = 0°-20°; **b** EICP-WFR, *α* = 0°-20°; **c** *α* = 30°.

**Fig. 14:**
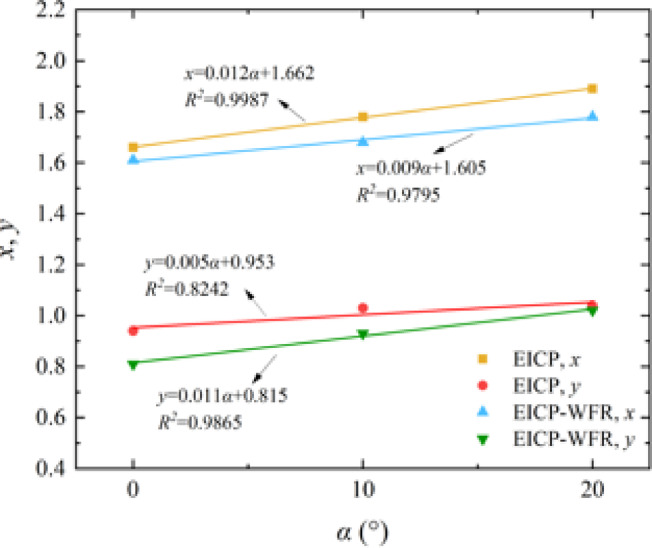
Parameter acquisition of erosion modulus model for solidified aeolian sand.

Figures 13 and 14 show the establishment and parameter acquisition of the wind erosion modulus model for solidified aeolian sand considering the influence of wind velocity and erosion angle. Table 3 lists the values of each parameter.

**Table 3:** Parameter values of erosion modulus model for solidified aeolian sand.

α (°)	Sample	x(x’)	y(c)	*R* ^2^
0	EICP	1.66	0.94	0.9767
	EICP-FR	1.61	0.81	0.9645
10	EICP	1.78	1.03	0.9844
	EICP-FR	1.68	0.93	0.9807
20	EICP	1.89	1.04	0.9887
	EICP-FR	1.78	1.02	0.9873
30	EICP	12.47	-22.60	0.9622
	EICP-FR	8.98	-18.18	0.8930

#### Model validation

**Fig. 15:**
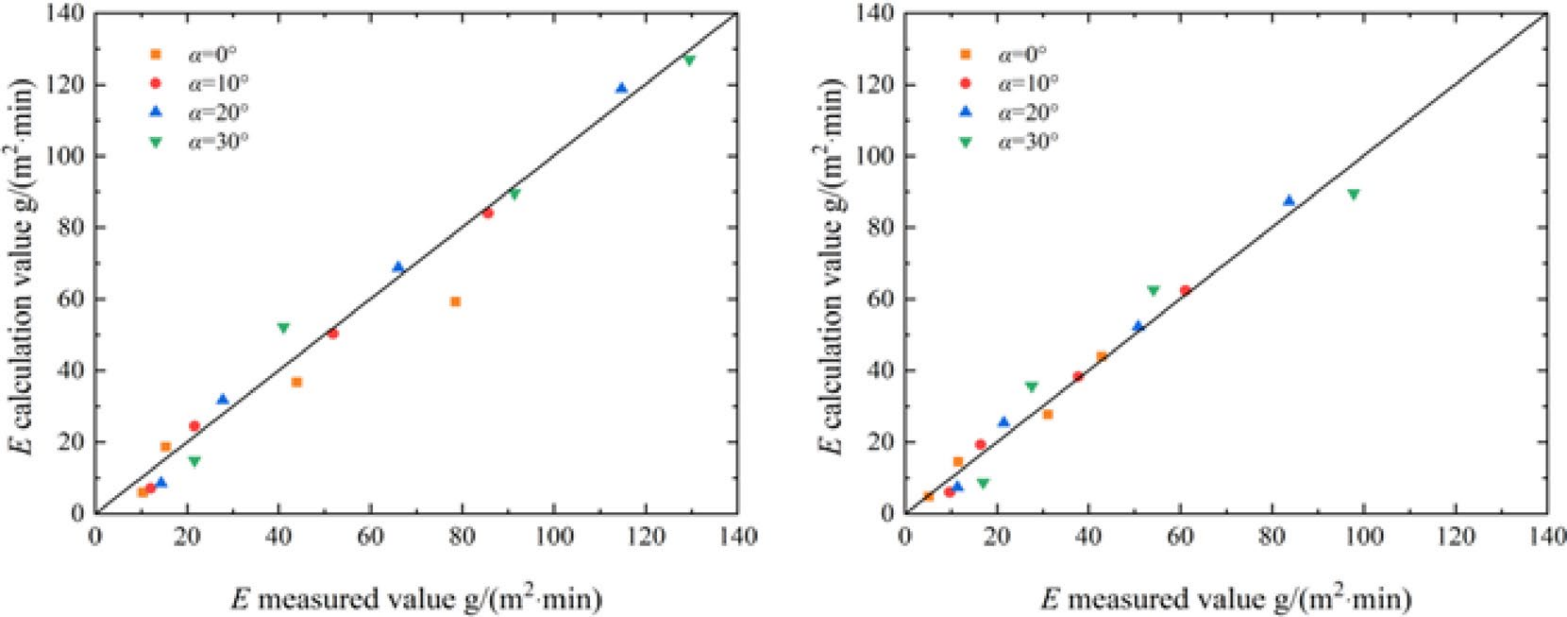
Comparison of measured and calculated values of *E* under wind erosion: **a** EICP; **b** EICP-FR.

Substitute the existing data *v*, *α*, as well as the obtained parameters *x*, *y*, *x’*, *c*, *x*_1_, *y*_1_, *c*_1_, *c*_2_ into Eq. (5), and calculate the wind erosion modulus *E* of the aeolian sand table solidified by EICP and EICP-WFR. Figure 15 shows the comparison between the calculated and measured values of the wind erosion modulus of the aeolian sand solidified by EICP and EICP-WFR. As shown in the figure, the fitted data is relatively evenly distributed on both sides of the bisector, indicating that the calculated results of the model are in good agreement with the experimental results, which verifies the reliability of the model.

## Conclusions

This article investigates the effects of wind velocity, erosion angle, and erosion cycles on the solidification of loose sand, EICP, EICP-BFR, and EICP-WFR sand through wind erosion experiments. Based on the experimental results, an erosion modulus model was established and validated for EICP and EICP synergistic fiber reinforcement solidification. The main conclusions are as follows:

1. Loose aeolian sand is almost completely eroded, while the EICP solidified sand forms a hard layer on the surface, retaining more sand and demonstrating its ability to resist wind erosion. EICP and fiber-reinforced solidified sand were partially blown away by wind, but the overall structure was not damaged, effectively protecting the uncured bottom layer of sand.
2. The wind velocity increases, and the rate of mass loss of aeolian sand accelerates. When the wind velocity increases from 3 m/s to 12 m/s, the mass loss rate of the scattered sand is between 2.36% and 88.79%, the mass loss rate of the aeolian sand solidified by EICP is between 1.46% and 63.55%, the mass loss rate of sand solidified by EICP-BFR is between 0.82% and 55.57%, and the mass loss rate of sand solidified by EICP-WFR is between 0.71% and 52.40%.
3. When the erosion angle increases from 0° to 30°, the mass loss rate of aeolian sand also increases, and shows a stepwise upward trend. The mass loss from 0° to 10° of erosion angle is relatively gentle, and the wind force cannot damage the solid surface with a smaller angle. The fluctuation from 10° to 30° is greater.
4. The frequency and angle of erosion increase, leading to an increase in the rate of quality loss. As the number of erosion cycles increases, the *η* of aeolian sand solidified by EICP increases from 1.46 to 7.59%, the *η* of aeolian sand solidified by EICP-BFR increases from 0.82 to 6.41%, and the *η* of aeolian sand solidified by EICP-WFR increases from 0.71 to 6.26%.
5. The aeolian sand solidified by EICP-BFR and EICP-WFR relies on the pulling of fibers, which reduces the separation and abrasion of small groups of sand particles and small blocks. The thickness of consolidation layer increases after adding fiber, which proves that the addition of fiber can effectively promote the cementation of CaCO_3_ crystal, improve the surface strength and wind erosion resistance, and reduce the quality loss of aeolian sand.
6. Based on the wind erosion test results, an erosion modulus model considering wind velocity and erosion angle was established and validated for the aeolian sand reinforced with EICP and EICP synergistic fiber reinforcement. The experimental results were in good agreement with the model calculation results, verifying the reliability of the regression model.
7. Notable findings from this study reveal that fiber reinforcement significantly enhances the performance of EICP-solidified aeolian sand: compared to EICP alone, the incorporation of wool fibers (EICP-WFR) increases the thickness of the consolidation layer by 62.5% and reduces mass loss by 17.5% under extreme erosion conditions. Practically, this EICP-fiber synergistic technology shows promising applicability for rapid wind erosion mitigation in arid desert regions characterized by loose, poorly graded sand deposits. However, the research has inherent limitations that warrant further investigation, including the long-term durability of the solidified layer under cyclic wet-dry environmental fluctuations and the scalability of the method for stabilizing large-scale sand dunes.

## Data Availability

All data generated or analyzed during this study are included in the published paper. The detailed data could be supplied on demand after corresponding author.
